# Notch Activation by Phenethyl Isothiocyanate Attenuates Its Inhibitory Effect on Prostate Cancer Cell Migration

**DOI:** 10.1371/journal.pone.0026615

**Published:** 2011-10-24

**Authors:** Su-Hyeong Kim, Anuradha Sehrawat, Kozue Sakao, Eun-Ryeong Hahm, Shivendra V. Singh

**Affiliations:** Department of Pharmacology and Chemical Biology, and University of Pittsburgh Cancer Institute, University of Pittsburgh School of Medicine, Pittsburgh, Pennsylvania, United States of America; University of Nebraska Medical Center, United States of America

## Abstract

Phenethyl isothiocyanate (PEITC) is a promising cancer chemopreventive component of edible cruciferous vegetables with *in vivo* efficacy against prostate cancer in experimental rodents. Cancer chemopreventive response to PEITC is characterized by its ability to inhibit multiple oncogenic signaling pathways, including nuclear factor-κB, Akt, and androgen receptor. The present study demonstrates, for the first time, that PEITC treatment activates Notch signaling in malignant as well as normal human prostate cells. Exposure of human prostate cancer cells (LNCaP, PC-3, and DU145) and a normal human prostate epithelial cell line (PrEC) to PEITC resulted in cleavage (active form) of Notch1 and Notch2, and increased transcriptional activity of Notch. In PC-3 and LNCaP cells, PEITC treatment caused induction of Notch ligands Jagged1 and Jagged2 (PC-3), overexpression of γ-secretase complex components Presenilin1 and Nicastrin (PC-3), nuclear enrichment of cleaved Notch2, and/or up-regulation of *Notch1*, *Notch2*, *Jagged1*, and/or *Jagged2* mRNA. PEITC-induced apoptosis in LNCaP and PC-3 cells was significantly attenuated by RNA interference of Notch2, but not by pharmacological inhibition of Notch1. Inhibition of PC-3 and LNCaP cell migration resulting from PEITC exposure was significantly augmented by knockdown of Notch2 protein as well as pharmacological inhibition of Notch1 activation. Nuclear expression of cleaved Notch2 protein was significantly higher in PC-3 xenografts from PEITC-treated mice and dorsolateral prostates from PEITC-fed TRAMP mice compared with respective control. Because Notch signaling is implicated in epithelial-mesenchymal transition and metastasis, the present study suggests that anti-metastatic effect of PEITC may be augmented by a combination regimen involving a Notch inhibitor.

## Introduction

Practical and safe modalities for chemoprevention of prostate cancer are clinically attractive because of high mortality associated with this malignancy in American men [1]. Plant products, including constituents of fruits and vegetables, continue to attract attention for the discovery of novel cancer chemopreventive agents [2]. Phenethyl isothiocyanate (PEITC) is one such promising cancer chemopreventive agent abundant in edible cruciferous vegetables such as watercress [3]. Evidence for protective effect of cruciferous vegetables and their components, including PEITC, against prostate cancer derives from population-based observational studies as well as laboratory investigations [3]–[9]. For example, a population-based case-control study suggested an inverse association between intake of cruciferous vegetables and the risk of prostate cancer [4]. *In vivo* chemopreventive efficacy of PEITC against prostate cancer has now been established in a transgenic mouse model (**Tr**ansgenic **A**denocarcinoma of **M**ouse **P**rostate model; hereafter abbreviated as TRAMP) [5], [6]. Feeding of 3 µmol PEITC/g diet significantly decreased incidence as well as burden (affected area) of poorly-differentiated cancer in the dorsolateral prostate of TRAMP mice [6]. Cancer chemopreventive response to PEITC is not restricted to the prostate cancer as inhibition of chemical carcinogenesis or suppression of spontaneous cancer development of other sites (*e.g.*, lung, colon, and esophagus) by this dietary component has also been documented [7]–[9]. Furthermore, growth of subcutaneous prostate cancer xenografts in athymic mice was significantly retarded by administration of PEITC or its *N*-acetylcysteine conjugate [10]–[12]. Notably, oral PEITC administration augmented proapoptotic response to docetaxel *in vivo* in prostate cancer xenografts [13].

Safety, bioavailability, selectivity towards cancer cells, and ability to target multiple oncogenic pathways are desirable attributes of a clinically useful cancer chemopreventive agent. Research thus far indicates that PEITC meets all these criteria. Firstly, PEITC is well-tolerated by experimental rodents [6]–[9]. Secondly, pharmacokinetic determinations indicate excellent bioavailability of PEITC [14], [15]. Thirdly, PEITC also shows selectivity towards cancer cells in causing apoptosis and autophagy [11], [16], [17]. Finally, PEITC is capable of suppressing multiple oncogenic signaling pathways that are hyperactive in human prostate cancer [18], including nuclear factor-κB (NF-κB) [19], Akt [20], signal transducer and activator of transcription 3 (STAT3) [21], and androgen receptor [22].

The present study extends these observations [19]–[22] and examines the effect of PEITC treatment on activation of Notch1 and Notch2, which belong to a family of transmembrane receptors implicated in prostate cancer development and metastasis [23], using cultured human prostate cancer cells (LNCaP, PC-3, LNCaP−C4-2, and DU145), a normal human prostate epithelial cell line (PrEC), PC-3 xenografts from control and PEITC-treated mice [13], [16], and dorsolateral prostate from control and PEITC-fed TRAMP mice [6].

## Results

### PEITC treatment increases levels of cleaved Notch1 and Notch2 in prostate cancer cells

**Figure 1:**
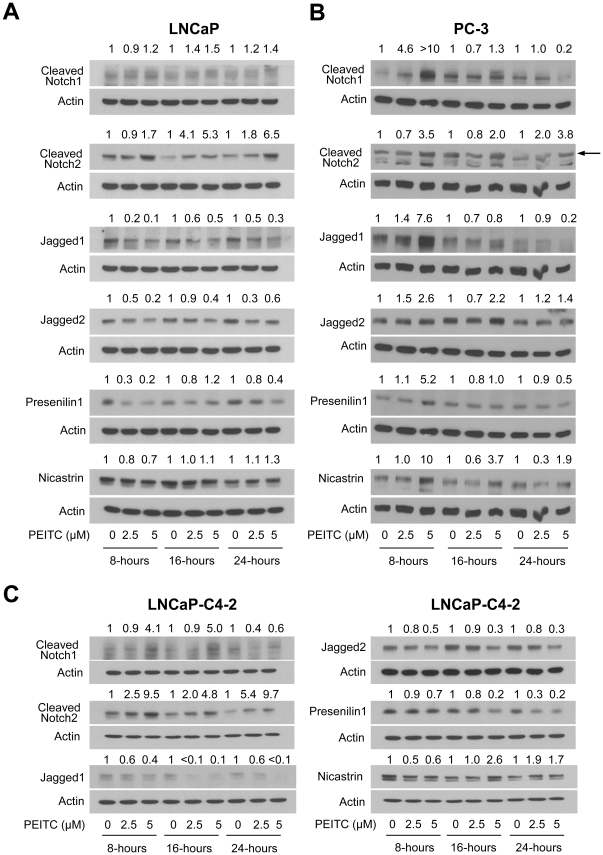
Phenethyl isothiocyanate (PEITC) increases levels of cleaved Notch1 and cleaved Notch2 in prostate cancer cells. Immunoblotting for cleaved Notch1, cleaved Notch2, Jagged1, Jagged2, Presenilin1, and Nicastrin using lysates from (A) LNCaP, (B) PC-3, and (C) LNCaP−C4-2 cells after 8-, 16-, or 24-hour treatment with dimethyl sulfoxide (DMSO) or PEITC (2.5 or 5 µM). Arrow in panel B identifies cleaved Notch2, the lower band is non-specific based on siRNA results shown in Fig. 4A. Blots were stripped and re-probed with anti-actin antibody. Immunoblotting for each protein was done at least twice using independently prepared lysates. Numbers above band represent changes in protein levels relative to corresponding DMSO-treated control.

Ligand-dependent activation of Notch is complex requiring cleavage by γ-secretase complex [23], [24]. Notch receptors are activated upon binding of their adjoining ligands (*e.g.*, Jagged1 and Jagged2), which is thought to induce a conformational change within the Notch receptor resulting in exposure of an S2 cleavage site for tumor necrosis factor-α converting enzyme [23], [24]. Subsequently, Notch receptors undergo another cleavage mediated by the γ-secretase complex at a site located within the Notch transmembrane domain [24]. Net outcome of this cleavage is the release of the Notch intracellular domain into the cytoplasm, which then translocates to the nucleus to regulate target gene expression [23], [24]. Level of cleaved Notch1 protein was increased upon treatment with PEITC in both LNCaP (Fig. 1A) and PC-3 cells (Fig. 1B) albeit with different kinetics and intensity. To the contrary, PEITC treatment caused a robust and sustained increase in the level of cleaved Notch2 protein in both LNCaP (Fig. 1A) and PC-3 cell lines (Fig. 1B) especially at the 5 µM dose. Based on Notch2 RNA interference data shown later, the lower band in the Notch2 western blot shown in Fig. 1B is non-specific. Effect of PEITC treatment on Jagged1 and Jagged2 protein expression was different between LNCaP and PC-3 cells. PEITC-treated LNCaP cell line generally exhibited a decrease in the levels of Jagged1 and Jagged2 proteins (Fig. 1A). In sharp contrast to LNCaP, transient (Jagged1) or sustained (Jagged2) induction of Jagged protein expression was clearly visible in PEITC-treated PC-3 cells (Fig. 1B). Differential responses were also discernible concerning effect of PEITC treatment on Presenilin1 and Nicastrin proteins between LNCaP and PC-3 cells especially at the 8-hour time point.

PC-3 cell line, which is androgen-independent, is relatively more aggressive compared with androgen-responsive LNCaP cells with respect to proliferation, *in vivo* growth in xenograft model, and cell migration. We questioned if differential response of LNCaP *versus* PC-3 cells to PEITC-mediated alterations in Notch signaling components was related to androgen-independent phenotype. We addressed this question using an androgen-independent variant of LNCaP cells (LNCaP−C4-2). Response of LNCaP−C4-2 cells to PEITC-mediated changes in Notch signaling proteins was generally similar to that observed in the parental LNCaP cells (Fig. 1C). Together, these observations indicated that while PC-3 and LNCaP cells differentially responded to PEITC-mediated changes in Notch ligands and γ-secretase complex, cleavage of Notch1 and Notch2 proteins upon PEITC exposure was consistent in each cell line tested. Also, transition of LNCaP cells to androgen-independence (LNCaP−C4-2) does not have any meaningful impact on PEITC-mediated changes in levels of Notch signaling components.

### PEITC treatment increases transcriptional activity of Notch

**Figure 2:**
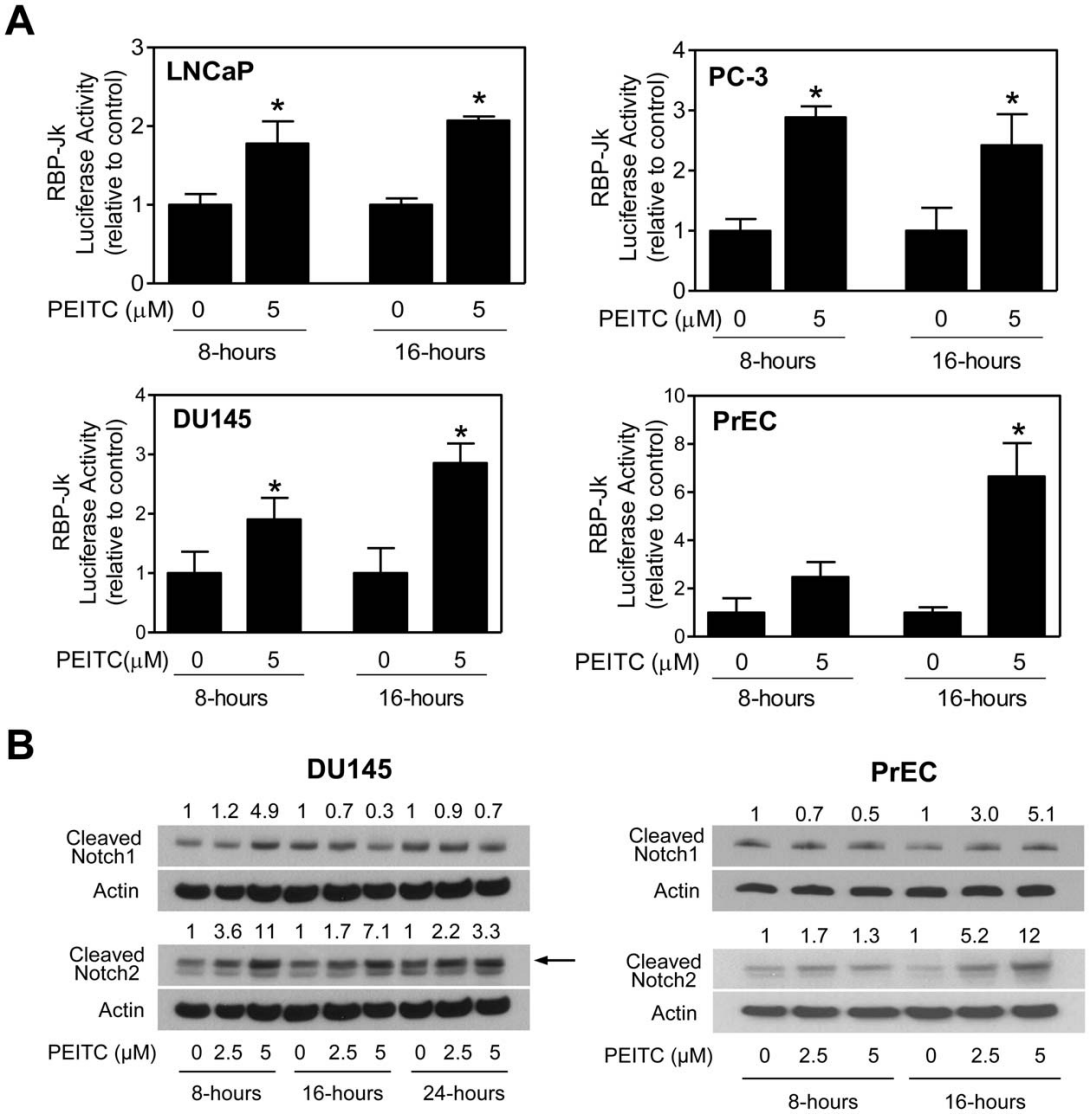
Phenethyl isothiocyanate (PEITC) treatment increases transcriptional activity of Notch in malignant and normal prostate cells. (A) Effect of PEITC treatment on RBP-Jk luciferase reporter activity (a measure of transcriptional activity of Notch) in LNCaP, PC-3, DU145, and PrEC cells after 8- or 16-hour treatment with dimethyl sulfoxide (DMSO) or 5 µM PEITC. Results shown are mean ± SD (n = 3). *Significantly different (*P*<0.05) compared with DMSO-treated control by one-way ANOVA followed by Dunnett's test (LNCaP and PC-3) or Student's t-test (DU145 and PrEC). (B) Immunoblotting for cleaved Notch1 and cleaved Notch2 using lysates from DU145 and PrEC cells treated with DMSO (control) or PEITC (2.5 or 5 µM) for the indicated time periods. Blots were stripped and re-probed with anti-actin antibody. Arrow in panel B (DU145 cells) identifies cleaved Notch2, the lower band is non-specific based on siRNA results shown in Fig. 4A. Immunoblotting for each protein was done at least twice using independently prepared lysates. Numbers above bands represent changes in protein levels relative to corresponding DMSO-treated control. Each experiment was repeated at least twice.

We questioned whether PEITC-mediated cleavage of Notch1 and Notch2 translated into increased transcriptional activity of Notch. As shown in Fig. 2A, treatment of LNCaP and, PC-3 cells with 5 µM PEITC resulted in a statistically significant increase in luciferase reporter activity of RBP-Jk (a downstream modulator of Notch signaling) compared with dimethyl sulfoxide (DMSO)-treated controls. We used another well-characterized castration-resistant human prostate cancer cell line (DU145) to determine the effect of PEITC treatment on transcriptional activity of Notch. As can be seen in Fig. 2A, PEITC treatment increased RBP-Jk luciferase reporter activity in DU145 cells as well. In addition, PEITC-treated DU145 cells exhibited similar kinetics of Notch1 and Notch2 cleavage (Fig. 2B) as observed in the PC-3 cell line (Fig. 1B).

Next, we used a normal human prostate epithelial cell line (PrEC) to determine if PEITC-mediated activation of Notch1 and Notch2 was unique to cancerous prostate cells. This was a worthy research objective considering striking differences have been noted with regards to effect of PEITC between cancerous and normal prostate cells. For example, we have shown previously that the PrEC cell line is significantly resistant to PEITC-mediated inhibition of oxidative phosphorylation, reactive oxygen species generation, and apoptosis induction compared with PC-3 and LNCaP cells [11], [17]. Furthermore, PC-3 and PrEC cells respond differentially to PEITC-mediated changes in expression of antioxidant defense genes [25]. Similar to prostate cancer cells (Fig. 1), PEITC treatment resulted in increased levels of cleaved Notch1 and Notch2 in PrEC cells especially at the 16-hour time point at both 2.5 and 5 µM concentrations (Fig. 2B). Consistent with results, PEITC-mediated increase in RBP-Jk luciferase reporter activity was also observed in PrEC cells after 16-hour treatment with 5 µM PEITC (Fig. 2A). Based on these results, we conclude that PrEC and cancerous prostate cells (PC-3, LNCaP, LNCaP−C4-2, and DU145) behave similarly with respect to PEITC-mediated activation of Notch.

### Effect of PEITC treatment on nuclear levels of cleaved Notch2

**Figure 3:**
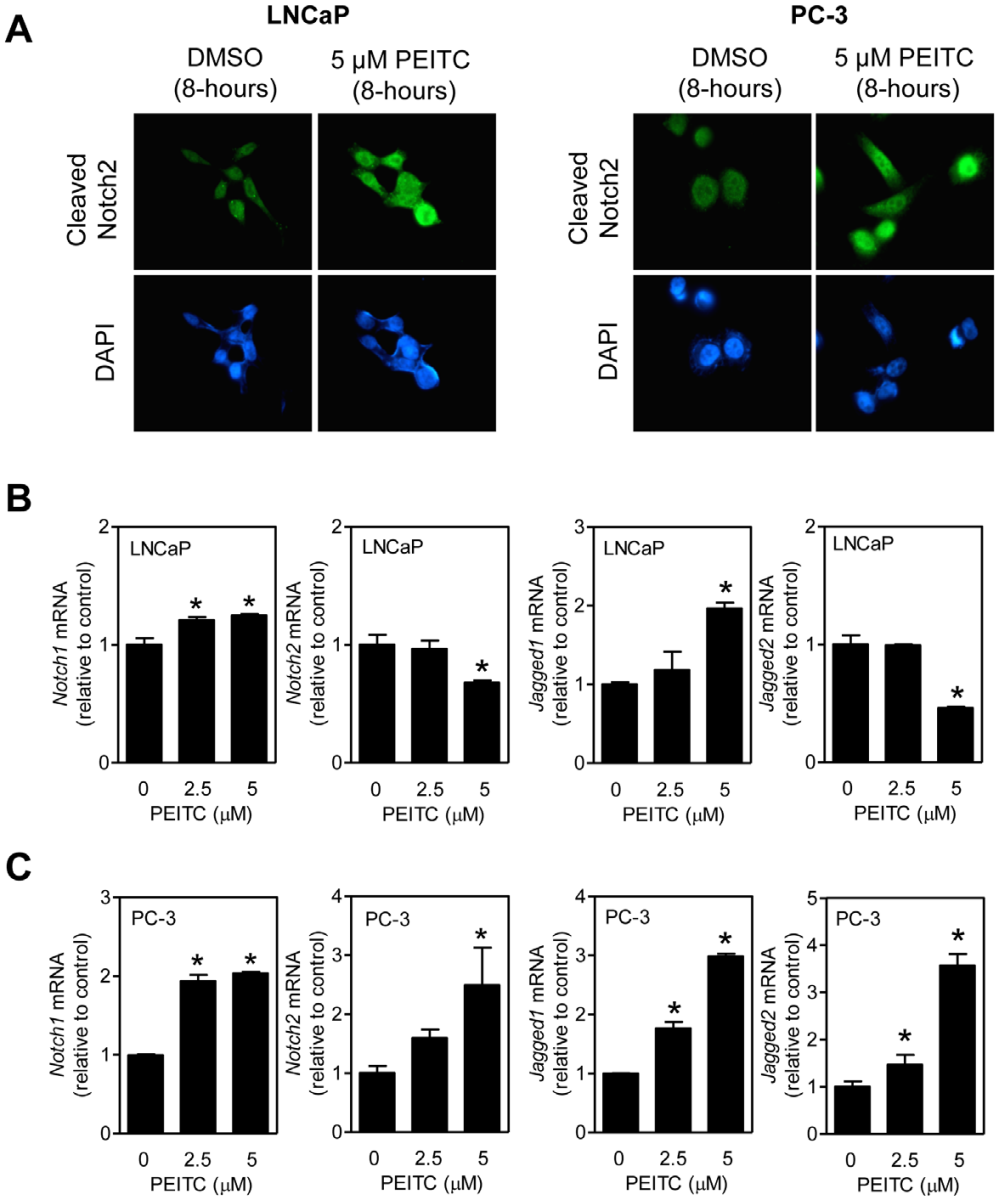
Phenethyl isothiocyanate (PEITC) increases nuclear levels of cleaved Notch2 protein in LNCaP and PC-3 cells. (A) Immunofluorescence microscopic images depicting nuclear levels of cleaved Notch2 protein in LNCaP and PC-3 cells after 8-hour treatment with dimethyl sulfoxide (DMSO) or 5 µM PEITC at 100× objective magnification. Quantitation of *Notch1*, *Notch2*, *Jagged1*, and *Jagged2* mRNA levels by real-time RT-PCR in (B) LNCaP and (C) PC-3 cells after 8-hour treatment with DMSO (control) or PEITC (2.5 or 5 µM). Results shown are mean ± SD (n = 3). *Significantly different (*P*<0.05) compared with DMSO-treated control by one-way ANOVA with Dunnett's adjustment. Each experiment was repeated at least twice.

Because the effect of PEITC treatment was relatively more pronounced and sustained on Notch2 cleavage compared with cleaved Notch1, we proceeded to determine cleaved Notch2 levels in DMSO-treated control and PEITC-treated LNCaP and PC-3 cells. PEITC-treated LNCaP and PC-3 cells exhibited a marked increase in the nuclear levels of cleaved Notch2 in comparison with DMSO-treated control (Fig. 3A). These results indicated that PEITC treatment resulted in nuclear enrichment of cleaved Notch2 in prostate cancer cells, which is consistent with the observed increase in transcriptional activity of Notch by PEITC treatment (Fig. 2A).

### Effect of PEITC treatment on *Notch1*, *Notch2*, *Jagged1*, and *Jagged2* mRNA levels

Data for the effect of PEITC treatment on mRNA levels of *Notch1*, *Notch2*, *Jagged1*, and *Jagged2* are shown in Fig. 3B (LNCaP) and Fig. 3C (PC-3). Expression of *Notch1* (2.5 and 5 µM PEITC) and *Jagged1* (5 µM PEITC) mRNA was increased significantly upon 8-hour treatment of LNCaP cells with PEITC (Fig. 3B). A similar PEITC treatment resulted in suppression of *Notch2* (5 µM PEITC) and *Jagged2* (5 µM PEITC) mRNA levels in LNCaP cells (Fig. 3B). On the other hand, PC-3 cells treated for 8 hours with 5 µM PEITC exhibited significant induction of *Notch1*, *Notch2*, *Jagged1*, and *Jagged2* mRNA expression compared with DMSO-treated control (Fig. 3C). Significant induction of *Notch1*, *Jagged1*, and *Jagged2* mRNA with 2.5 µM PEITC treatment was also observed in PC-3 cells (Fig. 3C). Once again, these results pointed towards cell line-specific differences in PEITC-mediated alterations in expression of *Notch1*, *Notch2*, *Jagged1*, and *Jagged2* mRNA.

### RNA interference of Notch2 confers protection against PEITC-induced apoptosis

**Figure 4:**
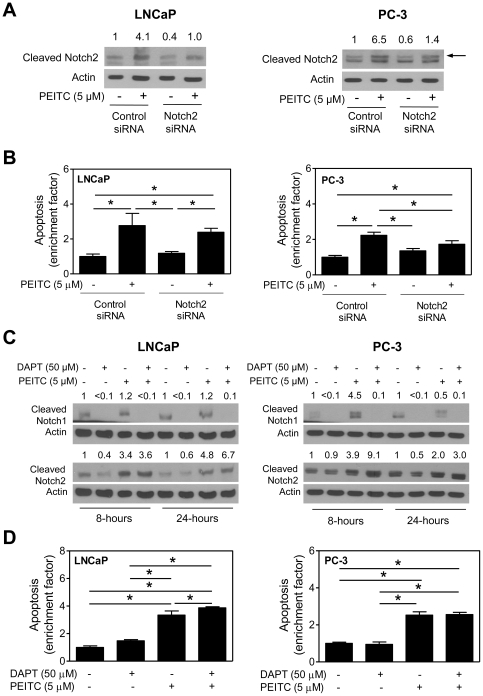
Notch2 knockdown confers protection against phenethyl isothiocyanate (PEITC)-induced apoptosis in LNCaP and PC-3 cells. (A) Immunoblotting for cleaved Notch2 protein using lysates from LNCaP and PC-3 cells transiently transfected with a control (non-specific) siRNA or a Notch2-targeted siRNA and treated for 24 hours with dimethyl sulfoxide (DMSO) or 5 µM PEITC. Numbers above bands represent changes in protein levels relative to control siRNA-transfected cells treated with DMSO. Arrow (PC-3) identifies cleaved Notch2, the lower band is non-specific. (B) Histone-associated DNA fragment release into the cytosol (a measure of apoptosis) in LNCaP and PC-3 cells transiently transfected with a control siRNA or a Notch2-targeted siRNA and treated for 24 hours with DMSO (control) or 5 µM PEITC. Results are expressed as apoptosis enrichment relative to control siRNA-transfected cells treated with DMSO. (C) Immunoblotting for cleaved Notch1 and cleaved Notch2 proteins using lysates from LNCaP and PC-3 cells treated for 8 hours or 24 hours with DMSO (control) or 5 µM PEITC in the absence or presence of 50 µM DAPT. Numbers above bands represent changes in protein levels relative to corresponding DMSO-treated control. (D) Histone-associated apoptotic DNA fragment release into the cytosol in LNCaP and PC-3 cells treated for 24 hours with DMSO (control) or 5 µM PEITC in the absence or presence of 50 µM DAPT. Results are expressed as apoptosis enrichment relative to DMSO-treated control. Results (panels B and D) are mean ± SD (n = 3). *Significantly different (*P*<0.05) between the indicated groups by one-way ANOVA followed by Bonferroni's multiple comparison test. Each experiment was repeated twice, and representative data from one such experiment are shown.

O'Neill et al [26] have shown previously that Notch2 regulates apoptosis in MDA-MB-231 cells. Because PEITC treatment consistently increased the levels of cleaved Notch2 protein in each cell line tested, it was only logical to determine if Notch2 contributed to PEITC-induced apoptosis. As shown in Fig. 4A, protein level of cleaved Notch2 was decreased by about 40–60% upon transient transfection of LNCaP and PC-3 cells with a Notch2-targeted small-interfering RNA (siRNA) in comparison with cells transfected with a control (non-specific) siRNA. PEITC-mediated increase in levels of cleaved Notch2 protein was clearly visible in control siRNA-transfected LNCaP and PC-3 cells, which was nearly fully abolished by RNA interference of Notch2 (Fig. 4A). Knockdown of Notch2 protein alone did not have any meaningful impact on histone-associated DNA fragment release into the cytosol, which is a well-accepted method for quantitation of apoptosis, in either cell line (Fig. 4B). On the other hand, PEITC-induced apoptosis was relatively more pronounced in LNCaP and PC-3 cells transfected with the control siRNA compared with those transfected with Notch2-targeted siRNA (Fig. 4B). These results indicated that Notch2 knockdown conferred protection against PEITC-induced apoptosis.

We designed experiments using a pharmacological inhibitor of γ-secretase {N-[N-(3,5-difluorophenacetyl-L-alanyl)]-S-phenylglycine-t-butyl ester; hereafter abbreviated as DAPT} to further test the role of Notch in PEITC-induced apoptosis. PEITC-mediated increase in levels of cleaved Notch1 protein, but not cleaved Notch2, was markedly suppressed by co-treatment with 50 µM DAPT (Fig. 4C). As expected, DAPT treatment alone decreased levels of cleaved Notch1 and Notch2 in both LNCaP and PC-3 cells, albeit to varying extent (Fig. 4C). PEITC-induced apoptosis was either not altered at all (PC-3 cells) or slightly increased (LNCaP cells) by co-treatment with DAPT (Fig. 4D). Based on these results, we conclude that activation of Notch2, but not Notch1, contributes to PEITC-induced apoptosis at least in PC-3 cells.

### RNA interference of Notch2 augments PEITC-mediated inhibition of cell migration

**Figure 5:**
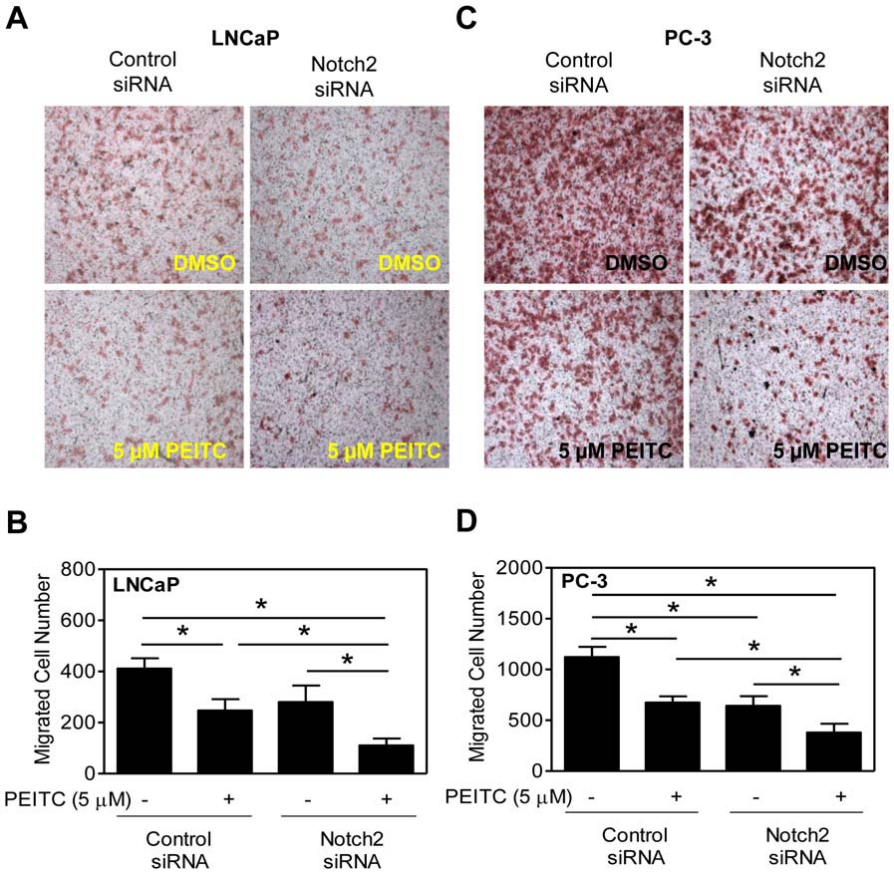
RNA interference of Notch2 augments phenethyl isothiocyanate (PEITC)-mediated inhibition of LNCaP and PC-3 cell migration. Representative images (Boyden chamber assay) depicting migration of (A) LNCaP and (C) PC-3 cells transfected with a control (non-specific) siRNA or a Notch2-targeted siRNA and treated for 24 hours with dimethyl sulfoxide (DMSO) or 5 µM PEITC at 200× magnification. Quantitation of migrated (B) LNCaP cells and (D) PC-3 cells from experiments shown in panels A and C. Three to four fields on each filter were scored for migrated cells under an inverted microscope at 200× magnification. Results shown are mean ± SD (n = 3). *Significantly different (*P*<0.05) between the indicated groups by one-way ANOVA followed by Bonferroni's multiple comparison test. Each experiment was repeated twice, and representative data from one such experiment are shown.

Because Notch signaling is implicated in cell invasion and epithelial-mesenchymal transition (EMT) [27], [28], we designed functional experiments to determine the consequences of Notch activation on PEITC's ability to inhibit LNCaP and PC-3 cell migration. Transient transfection with Notch2-targeted siRNA alone resulted in suppression of LNCaP (Fig. 5A) and PC-3 (Fig. 5C) cell migration compared with corresponding control siRNA-transfected cells as determined by Boyden chamber assay. Migration of control siRNA-transfected LNCaP (Fig. 5B) and PC-3 cells (Fig. 5D) was also reduced significantly upon treatment with 5 µM PEITC. Furthermore, PEITC-mediated inhibition of LNCaP (Fig. 5B) and PC-3 (Fig. 5D) cell migration was significantly augmented by knockdown of the Notch2 protein.

### Pharmacological suppression of Notch1 activation augments PEITC-mediated inhibition of cell migration

**Figure 6:**
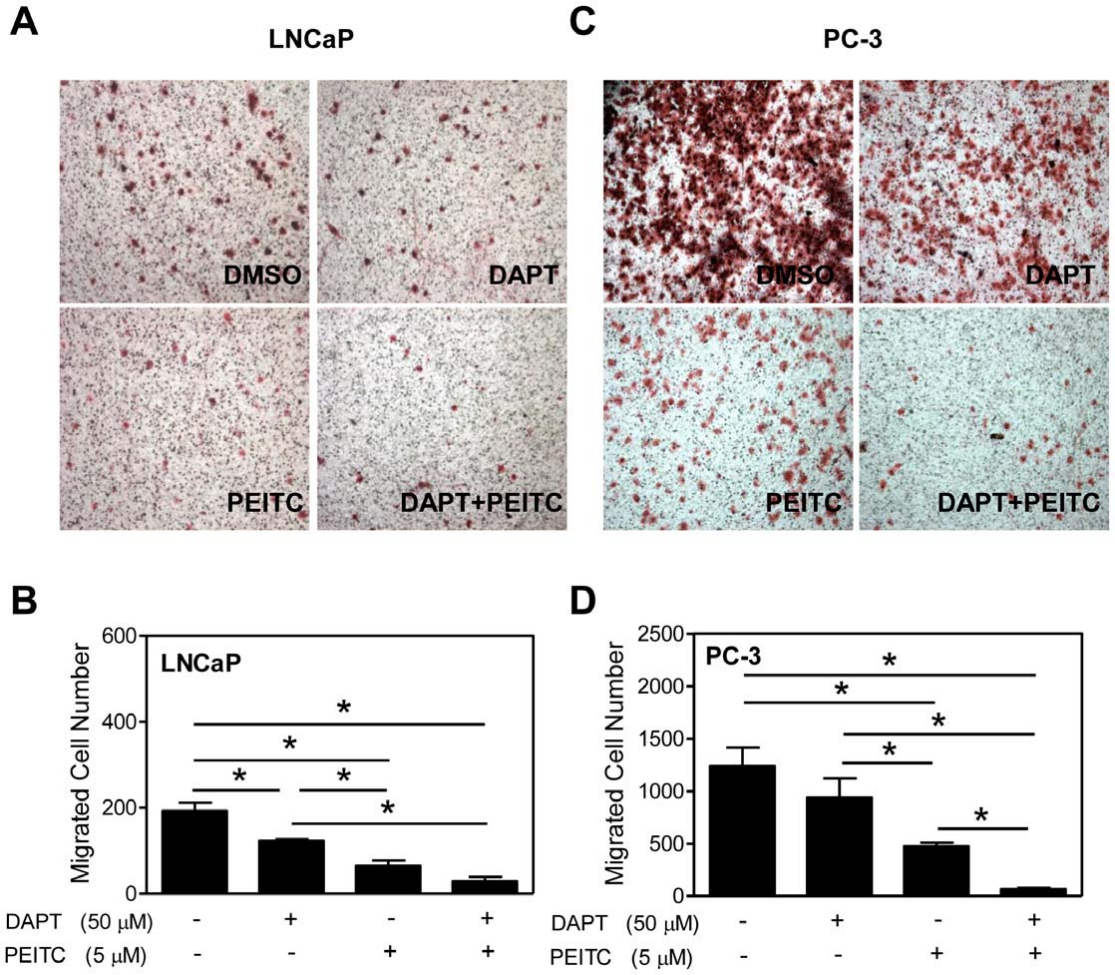
Phenethyl isothiocyanate (PEITC)-mediated inhibition of prostate cancer cell migration is augmented by a γ-secretase inhibitor. Representative images (Boyden chamber assay) depicting migration of (A) LNCaP and (C) PC-3 cells after 24-hour treatment with dimethyl sulfoxide (DMSO) or 5 µM PEITC in the absence or presence of 50 µM DAPT at 200× magnification. Quantitation of migrated (B) LNCaP cells and (D) PC-3 cells after 24-hour treatment with DMSO or 5 µM PEITC and/or 50 µM DAPT. Three to four fields on each filter were scored for migrated cells under an inverted microscope. Results shown are mean ± SD (n = 3). *Significantly different (*P*<0.05) between the indicated groups by one-way ANOVA followed by Bonferroni's multiple comparison test. Each experiment was repeated twice, and representative data from one such experiment are shown.

DAPT alone caused a modest decrease in LNCaP (Fig. 6A) and PC-3 (Fig. 6C) cell migration compared with respective DMSO-treated control. Similar to data using Notch2 siRNA, co-treatment with DAPT augmented PEITC-mediated inhibition of LNCaP (Fig. 6B) and PC-3 (Fig. 6D) cell migration. These results indicated that Notch1 and Notch2 activation by PEITC negatively impacts its ability to inhibit prostate cancer cell migration.

### Immunohistochemical analysis for the effect of PEITC on cleaved Notch2 protein *in vivo*

**Figure 7:**
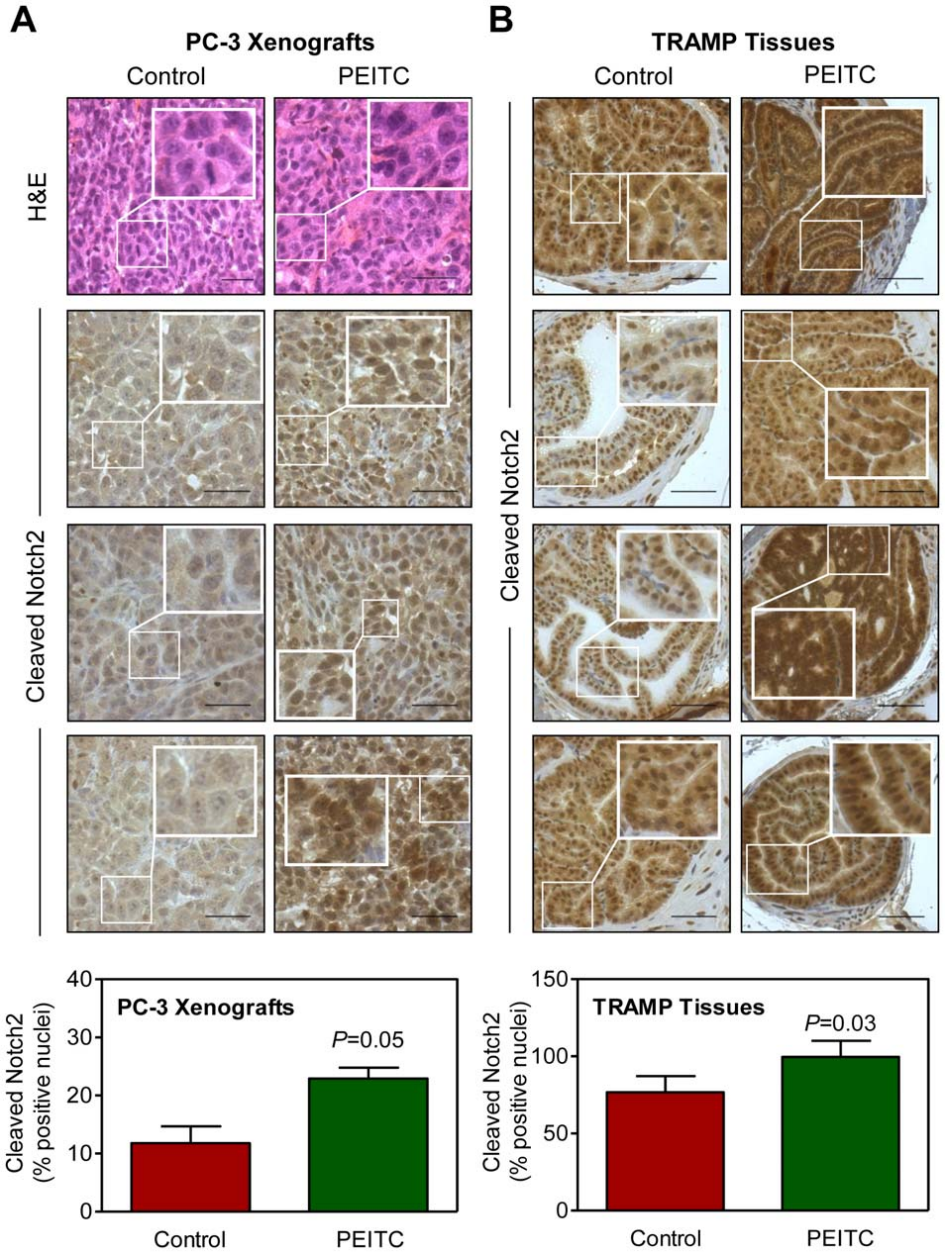
*In vivo* effect of phenethyl isothiocyanate on expression of cleaved Notch2 protein in prostate tumor. (A) Representative H&E staining and immunohistochemical staining for expression of cleaved Notch2 protein in PC-3 xenografts from three mice of the indicated groups (scale bar, 50 µm; magnification, 400×). Amplification of selected area is shown in the inset. Quantitation of nuclear expression of Notch2 protein in PC-3 xenografts from control and phenethyl isothiocyanate (PEITC)-treated mice is shown in the bar graph (mean ± SD; n = 3). (B) Immunohistochemistry showing cleaved Notch2 protein expression in the dorsolateral prostates from four TRAMP mice of the indicated groups (scale bar, 50 µm; magnification, 400×). Amplification of selected area is shown in the inset. Quantitation of nuclear expression of cleaved Notch2 protein in the dorsolateral prostates of control and PEITC-fed TRAMP mice is shown in the bar graph (mean ± SD; n = 4). Statistical significance was determined by Student's t-test.

We used archived tissues from our previously completed studies [6], [13], [16] to determine *in vivo* relevance of the cellular findings (Fig. 1). Because the effect of PEITC treatment was most consistent and sustained on cleaved Notch2, the immunohistochemical analysis was restricted to this protein. Representative immunohistochemical images for cleaved Notch2 expression in PC-3 tumor xenograft sections from control and PEITC-treated mice are shown in Fig. 7A. In agreement with the results shown in cultured PC-3 cells (Fig. 3A) nuclear expression of cleaved Notch2 was significantly higher in PC-3 xenografts from PEITC-treated mice compared with control (Fig. 7A). Similarly, the nuclear level of cleaved Notch2 protein in the dorsolateral prostate was significantly higher in PEITC-fed TRAMP mice compared with control (Fig. 7B).

## Discussion

Precise role of Notch signaling in prostate cancer development is still unclear, but studies have tried to resolve this issue with the use of prostate cancer cell lines and human prostate cancer biopsies. Down-regulation of Jagged1 has been shown to inhibit proliferation of prostate cancer cells [29]. The same group of investigators reported later that RNA interference of Notch1 conferred protection against prostate cancer cell migration and invasion [30]. At the same time, overexpression of constitutively active Notch1 has also been shown to inhibit proliferation of LNCaP cells [31]. Because Notch signaling is quite complex involving interplay between four receptors (Notch1-Notch4) and five ligands [Jagged1, Jagged2, Delta-like ligands (Dll1, Dll3, and Dll4)] [23], [24] and each component of Notch signaling is not commonly studied [29]–[31], it is plausible that the discrepancy stems from compensatory changes in other components triggered by knockdown of Notch1 or Jagged1. Nevertheless, Jagged1 expression in prostate cancer biopsies is associated with increased metastasis and recurrence [32]. The present study reveals that PEITC activates Notch1 and Notch2 in cancerous and normal prostate cells. Moreover, PEITC administration causes significant increase in nuclear levels of cleaved Notch2 *in vivo* in prostate tumors from two different rodent models. We also demonstrate that activation of Notch1 and Notch2 reduces PEITC's ability to inhibit prostate cancer cell migration. Based on these observations, we are tempted to speculate that anti-metastatic effect of PEITC may be augmented by a combination regimen involving a Notch inhibitor.

LNCaP and PC-3 cells exhibit striking differences with regards to PEITC-mediated alterations in Notch signaling components, especially Notch ligands and γ-secretase components. Two obvious possibilities deserve attention to explain these differences in PEITC response between androgen-responsive LNCaP cells (wild-type p53) *versus* castration-resistant and p53-null PC-3 cells. One such possibility relates to difference in status of p53 between LNCaP and PC-3 cells as Notch1 has been shown to be a direct target of p53 [33]. Restoration of p53 expression in human cancer cell lines has been shown to up-regulate the expression of Notch1 [33]. Because PC-3 and DU145 cells, both of which lack functional p53, behave similarly with regards to PEITC-mediated activation of Notch1 and Notch2, possibility that differential response between LNCaP and castration-resistant cells (PC-3 and DU145) is manifested, partially if not completely, by the p53 can't be discarded at present. At the same time, we are aware that the regulation of expression as well as activation of Notch1 is quite complex mediated by other factors besides p53, including Nrf2, ETS transcription factor family member PEA3, epidermal growth factor receptor signaling, and Akt to name a few [34]–[37]. While further studies are needed to resolve this question surrounding p53, it is reasonable to conclude that the differential response between LNCaP and PC-3 cells is not related to androgen-responsiveness because LNCaP cells and its androgen-independent variant LNCaP−C4-2 exhibit generally comparable response to PEITC-mediated changes in Notch signaling components. In this context, it is important to mention that *Notch* target HEY (Hairy/Enhancer of split-related with YRPW motif) is an androgen receptor selective co-repressor [38].

Activation of Notch2 by overexpression of its intracellular domain promotes apoptosis in MDA-MB-231 cells [26]. It was of interest to determine the consequences of Notch activation on proapoptotic response to PEITC in our model. Unlike Notch1 [30], a 40–60% knockdown of Notch2 protein has no meaningful impact on apoptosis in LNCaP or PC-3 cells. However, apoptosis resulting from PEITC exposure is significantly attenuated by Notch2 knockdown at least in PC-3 cells. On the other hand, suppression of Notch1 activation with DAPT has no effect on proapoptotic response to PEITC. Based on these results we conclude that Notch1 is dispensable for cell death resulting from PEITC exposure in PC-3 and LNCaP cells. Mechanism by which Notch2 knockdown confers protection against PEITC-induced apoptosis is not yet clear. Inhibition of several anti-apoptotic pathways, such as NF-κB and STAT3, coupled with alterations in expression of Bcl-2 family proteins have been implicated in apoptosis induction by PEITC [10], [11], [17], [19], [39]. It is possible that Notch2 knockdown alters effect of PEITC on some of these pathways to confer protection against apoptosis.

Metastasis is the primary cause of mortality from prostate cancer. Notably, Notch has been implicated in EMT [27], which promotes metastasis [40]. Moreover, treatment of LNCaP and PC-3 cells with γ-secretase inhibitor DAPT has been shown to inhibit cell motility [41]. The present study reveals that motility of both LNCaP and PC-3 cells is reduced significantly by knockdown of Notch2 protein using siRNA (decreased but not significant in LNCaP) as well as DAPT-mediated inhibition of Notch1 activation (reduced but not significant in PC-3). Furthermore, PEITC-mediated inhibition of LNCaP and PC-3 cell migration is augmented by knockdown of Notch2 protein as well as pharmacological inhibition of Notch1 cleavage (PC-3 only). Mechanism by which Notch2 knockdown inhibits cell migration remains elusive, but this issue has been studied for Notch1 [28]. Targeted depletion of Notch1 inhibits PC-3 and 22Rυ1 cell migration by down-regulating expression of matrix metalloproteinase-9 and urokinase plasminogen activator [28]. It is possible that Notch2 knockdown elicits similar response on matrix metalloproteinase-9 and urokinase plasminogen activator.

In conclusion, the present study demonstrates that PEITC treatment promotes cleavage of Notch1 and Notch2 leading to their transcriptional activation in both cancerous (LNCaP, PC-3, and DU145) and normal (PrEC) prostate cells. PEITC-induced apoptosis in prostate cancer cells is modestly attenuated by knockdown of Notch2, but not by pharmacological inhibition of Notch1 activation. On the other hand, targeted depletion of Notch2 by siRNA as well as inhibition of Notch1 activation using DAPT augments prostate cancer cell motility inhibition by PEITC.

## Methods

### Ethics statement

We used archived tissue sections from our previously published *in vivo* studies [6], [13], [16] to determine the effect of PEITC administration on expression of cleaved Notch2. Use of mice and their care for these studies [6], [13], [16] was approved by and in accordance with the University of Pittsburgh Institutional Animal Care and Use Committee guidelines.

### Reagents

PEITC (purity >98%) was purchased from LKT Laboratories (St. Paul, MN). Cell culture reagents were purchased from Invitrogen-Life Technologies (Carlsbad, CA). Antibodies used in the present study were purchased from the following sources: antibodies against cleaved Notch1, Presenilin1, Jagged1, Jagged2, and Nicastrin were from Cell Signaling Technology (Danvers, MA); an antibody specific for detection of cleaved Notch2 was from EMD-Millipore (Billerica, MA); and anti-actin antibody was from Sigma-Aldrich (St. Louis, MO). A γ-secretase inhibitor (DAPT) was purchased from Calbiochem (Billerica, MA). Notch2-targeted siRNA was purchased from Santa Cruz Biotechnology (Santa Cruz, CA). A non-specific control siRNA was purchased from Qiagen (Germantown, MD).

### Cell lines

LNCaP, PC-3, and DU145 human prostate cancer cell lines were obtained from American Type Culture Collection (Manassas, VA), and maintained as described by us previously [10], [13], [42], [43]. PrEC cells were purchased from Clonetics (SanDiego, CA) and maintained as described by us previously [11]. Cell line authentication was done by Research Animal Diagnostic Laboratory (University of Missouri, Columbia, MO). The cells were last tested in February 2011 and found to be of human origin. Moreover, genetic profile for each cell line was consistent with those in the American Type Culture Collection database. LNCaP−C4-2 cell line (not authenticated by us) was obtained from UroCor and maintained as described by us previously [43].

### Immunoblotting

Stock solution of PEITC was prepared in DMSO and an equal volume of DMSO (final concentration 0.1%) was added to controls. After treatment with DMSO or PEITC, cells were collected and processed for immunoblotting as described by us previously [6], [44].

### Luciferase reporter assay

Cignal RBP-Jk luciferase reporter kit from SABiosciences-Qiagen was used to determine the effect of PEITC treatment on transcriptional activity of Notch. The RBP-Jk [C protein binding factor 1/Suppressor of Hairless/Lag1 (CBF1/Su (H)/Lag 1)] is a direct downstream modulator of Notch signaling [23], [24]. Notch intracellular domain (cleaved Notch) binds to CSL/CBF1/Su (H)/Lag 1 and converts it from a transcriptional repressor to a transcriptional activator [23], [24]. Desired cells were transfected with the reporter construct using Fugene6. Twenty-four hours after transfection, cells were treated with DMSO (control) or PEITC (5 µM) for 8 or 16 hours, and harvested using lysis buffer. Samples were centrifuged, and 20 µL aliquot was used for measurement of dual luciferase activity using a luminometer. Luciferase activity was normalized to protein concentration and expressed as a ratio of firefly luciferase to renilla luciferase units.

### Immunofluorescence microscopy for cleaved Notch2

LNCaP or PC-3 cells (1×10^5^) were grown on coverslips in 12-well plates and allowed to attach by overnight incubation. Cells were treated with DMSO (control) or PEITC (5 µM) for 8 hours, fixed with 2% paraformaldehyde for 1 hour at room temperature, and permeabilized using 0.5% Triton X-100 for 5 minutes. Cells were then incubated with phosphate-buffered saline (PBS) supplemented with 0.5% bovine serum albumin and 0.15% glycine for 1 hour followed by overnight incubation with anti-cleaved Notch2 antibody (1∶500 dilution in above buffer) at 4°C. Next day, cells were treated with 2 µg/mL of Alexa Fluor-488-conjugated secondary antibody for 1 hour at room temperature. After washing with PBS, cells were treated with 50 ng/mL 4′,6-diamidino-2-phenylindole (DAPI) for 5 minutes at room temperature to stain nuclear DNA. Cells were mounted and visualized under a fluorescence microscope.

### Real-time reverse transcription-PCR (RT-PCR)

Total RNA from DMSO-treated control and PEITC-treated cells was isolated using RNeasy kit (Qiagen). First-strand cDNA was synthesized using Superscript reverse transcriptase (Invitrogen-Life Technologies) with oligo (dT)_20_ primer. Primers were as follows: *Notch1*: forward 5′-CACTGTGGGCGGGTCC-3′, reverse 5′-GTTGTATTGGTTCGGCACCAT-3′; *Notch2*: forward 5′-AATCCCTGACTCCAGAACG-3′, reverse 5′-TGGTAGACCAAGTCTGTGATGAT-3′; *Jagged1*: forward 5′-CTATGATGAGGGGGATGCT-3′, reverse 5′-CGTCCATTCAGGCACTGG-3′; *Jagged2*: forward 5′-TGGGATGCCTGGCACA-3′, reverse 5′-CCGGCAGATGCAGGA-3′; *GAPDH*: forward 5′-GGACCTGACCTGCCGTCTAGAA-3′, reverse 5′-GGTGTCGCTGTTGAAGTCAGAG-3′. Quantitative real-time RT-PCR was done using 2× SYBR Green master mix (Applied Biosystems-Life Technologies) with 55°C annealing (45 seconds for *Notch1* and *Notch2*), 53°C annealing (45 seconds for *Jagged1* and *Jagged2*) for 40 cycles. Relative gene expression was calculated using the method described by Livak and Schmittgen and normalized against *GAPDH* [45].

### RNA interference of Notch2

LNCaP or PC-3 cells were transfected at ∼50% confluency with 100 nmol/L of Notch2- targeted siRNA or control siRNA using Oligofectamine. Twenty-four hours after transfection, cells were treated with DMSO (control) or PEITC (5 µM) for 24 hours. Subsequently, cells were collected and processed for western blotting, quantitation of histone-associated DNA fragment release into the cytosol (a method for quantitation of apoptosis), and cell migration. Histone-associated DNA fragment release into the cytosol was quantified using Cell Death Detection ELISA kit (Roche Applied Science, Indianapolis, IN) by following the manufacturer's instructions.

### Cell migration assay

Transwell Boyden chambers containing 8.0 µm pore size polycarbonate filter (Corning-Life Sciences, Big Flats, NY) were used to determine cell migration. LNCaP or PC-3 cells were transfected with a control siRNA or Notch2-targeted siRNA as above, and used for cell migration assay described by us previously [20].

### Immunohistochemistry

Immunohistochemistry was performed essentially as described by us previously for other proteins [6], [16]. Three (xenografts) or five dorsolateral prostate specimens (TRAMP tissues) from different mice of each group were used. Data for one TRAMP mouse from both groups was not included in the analysis due to larger than usual variation. Multiple non-overlapping representative images from each section were captured using Image ProPlus 5.0 software. Expression of cleaved Notch2 in the nucleus was determined using Nuclear v9.1 algorithm of Aperio Image Scope software. This software automatically counts stained (brown color) nuclei and categorizes them according to intensity (0, 1+, 2+ or 3+). Results are computed as percent positive nuclei accounting for both nuclear count and intensity.
